# The impact of an educational podcast on emotional wellbeing and attitudes towards Charles Bonnet syndrome hallucinations

**DOI:** 10.1177/25158414261446576

**Published:** 2026-06-06

**Authors:** Bethany E. Higgins, Deanna J. Taylor, Sonali Dave, Sarah Sowerby, David P. Crabb, Tamsin Callaghan

**Affiliations:** Optometry and Visual Sciences, City St. George’s, Northampton Square, London, UK; Optometry and Visual Sciences, City St. George’s, Northampton Square, London, UK; Optometry and Visual Sciences, City St. George’s, Northampton Square, London, UK; WordBird, Battersea Studios, Nine Elms, London, UK; Optometry and Visual Sciences, City St. George’s, Northampton Square, London, UK; NIHR Royal Free Clinical Research Facility, Research and Development, Royal Free London NHS Foundation Trust, 02/62, Second Floor, Clinic Block, Royal Free Hospital, Pond Street, London NW3 2QG, UK

**Keywords:** Charles Bonnet syndrome, emotional well-being, hallucination management, intervention, podcast

## Abstract

**Background:**

Charles Bonnet Syndrome (CBS) is characterised by visual hallucinations in individuals with visual impairment, which can negatively impact emotional wellbeing. Educational interventions may help improve understanding and coping.

**Objective:**

This study investigated whether an educational podcast on CBS could enhance emotional wellbeing and promote more positive attitudes toward hallucinations.

**Design:**

Pre–post exploratory study.

**Methods:**

Participants were recruited via charities and social media as part of a larger study on the psychosocial impact of CBS. A baseline survey collected data on CBS symptom characteristics, the perceived impact of hallucinations (rated on a 5-point Likert scale from very negative to very pleasant) and level of negative affect (negative emotional wellbeing), assessed by the Positive and Negative Affect Schedule (PANAS). Participants then engaged with an educational podcast about CBS over a 3-week period. A follow-up survey assessed changes in emotional wellbeing, attitudes toward hallucinations, and symptom perception.

**Results:**

Fifty-four individuals with CBS (76% female; 70% aged >65 years) received the podcast. After the podcast intervention, 22 participants (41%) reported a less negative impact of CBS on their lives. There was a statistically significant improvement in perceived CBS impact (*p* < 0.01) and a statistically significant reduction in negative emotional wellbeing (*p* = 0.02).

**Conclusion:**

The educational podcast intervention was associated with improved emotional wellbeing, reflected in statistically significantly reduced negative affect and a decreased perceived burden of CBS. These findings suggest that educational podcasts may serve as a valuable tool for supporting the emotional health of individuals with CBS.

## Plain language summary

How an educational podcast helped people with Charles Bonnet syndrome feel better about their hallucinations

People with Charles Bonnet Syndrome (CBS) often experience visual hallucinations, which can sometimes feel confusing or upsetting. This study looked at whether listening to an educational podcast about CBS could help people feel more positive. Fifty-four people with CBS took part. Before the podcast, they answered questions about how their hallucinations affected them and how they were feeling emotionally. They then listened to the podcast over three weeks, and afterwards answered the same questions again. The results showed that many participants felt less negatively about their hallucinations after listening. On average, people reported that the hallucinations bothered them less, and they also showed an improvement in emotional well-being. This suggests that podcasts may be a helpful and accessible way to support people living with CBS.

## Introduction

Charles Bonnet Syndrome (CBS) is characterised by visual hallucinations in individuals with a visual impairment, but without a psychological cause. Alongside a visual impairment, advancing age and social isolation have been identified as contributing risk factors for CBS.^1,2^ CBS may also occur in patients following post-neurosurgical complications.^3^ Poignantly, a relatively large proportion of the visually impaired population (~20%) is expected to experience or have experienced symptoms of CBS.^4,5^ Yet, the psychological impact of CBS remains poorly understood. As visual hallucinations are associated with cognitive decline, it has been suggested the stigma surrounding symptoms of CBS results in hesitancy to disclose hallucinations to family, friends and medical professionals.

It is well established that visual impairment is an indicator of poorer quality of life (QOL).^6,7^ However, CBS has been identified as an additional factor contributing to lower self-reported QOL and emotional distress in individuals with visual impairment, even when visual functionality is accounted for.^8^ Furthermore, loneliness has been found to exacerbate CBS symptoms^9^ and people who report greater feelings of social isolation and negative emotional well-being were more likely to perceive CBS as having a detrimental impact on their life.^10^ Research suggests that approximately one-third of individuals with CBS report a significantly negative impact on their lives, a phenomenon termed ‘negative-outcome CBS’ by Cox and Ffytche.^11^ Given the profound effect of CBS on this subgroup, further research is needed to identify effective strategies for managing the condition.

Since QOL assessments are subjective and influenced by individual expectations, self-reported measures are inherently shaped by emotional states. Negative affect, including emotions such as dysphoria or anger, can influence how individuals perceive and report their health and overall wellbeing.^12^ This extends to how those with CBS interpret the impact of their visual hallucinations. Our previous findings demonstrate that negative affect is significantly associated with the perceived burden of CBS symptoms on daily life.^10^

Given the strong link between emotional wellbeing and the perceived impact of CBS, providing appropriate support is essential in helping individuals feel more in control of their symptoms. Educational resources that enhance understanding of CBS may empower individuals to manage their experiences more effectively. However, despite CBS affecting those with a visual impairment, information on management and support available is typically found in written format, either via websites or leaflets. Podcasts, as digital audio files that can be accessed on various devices,^13^ present a novel and cost-effective way to disseminate health information. They offer an accessible and convenient platform, particularly for visually impaired individuals. Research suggests that educational podcasts can improve knowledge, enhance self-efficacy and influence attitudes across diverse topics,^14^ while also proving valuable in promoting health-related behaviours.^15^

The objective of this study was to examine whether the emotional wellbeing of participants changed after receiving an education-focused podcast, utilising validated outcome measures such as the Positive and Negative Affect Schedule (PANAS) and participant-reported ratings of CBS impact on their lives. By assessing changes in both emotional state and perceived burden of symptoms, this study aimed to determine whether an accessible, audio-based educational intervention can serve as a meaningful tool in improving wellbeing and fostering a more positive outlook for individuals living with CBS.

## Methods

This pre–post exploratory intervention study was reported in accordance with the Transparent Reporting of Evaluations with Nonrandomised Designs (TREND) guidelines.^16^ A completed TREND checklist is provided in the Supplemental Material. Portions of the methodology and selected descriptive results have been published previously in a study comparing two closely related podcast interventions.^17^ These are included here to provide methodological context for the current analyses, which examine combined data from both podcast versions to address a distinct research objective.

### Patient and public involvement and engagement

Before the study was initiated, patient and public involvement and engagement (PPIE) activities were conducted to better understand the experiences of individuals living with hallucinations. This input was essential in refining the questionnaire design, ensuring its relevance and accessibility to the target population. In the development of the educational podcast, expertise was sought from individuals familiar with CBS and visual impairment. A psychiatrist specialising in CBS and a patient with CBS and a visual impairment contributed their perspectives, ensuring that the podcast content accurately reflected the lived experiences of those affected by the condition.

To further support accessibility, a focus group involving individuals with age-related macular degeneration (AMD) was held, providing valuable feedback on the questionnaire’s design and usability. In addition, the online surveys were reviewed by the charities Macular Society and Bravo Victor, who tested compatibility with widely used screen-reader technologies (e.g., NonVisual Desktop Access and Job Access with Speech). These checks confirmed that the questionnaires were screen-reader accessible. For participants who did not have computer access or were not comfortable completing the survey independently, a member of the research team (BH) was available to administer the questionnaire via telephone.

### Participant recruitment

Participants were recruited to this pre–post exploratory study using a convenience-based self-selection approach. Recruitment occurred via community-based online social media advertisements and with the assistance of national and local vision-related charities, including the Macular Society, Esme’s Umbrella, RNIB, and the Lincoln and Lindsey Blind Society (see Acknowledgements). This recruitment formed part of a larger study investigating the psychosocial impact of CBS.^10^ To be eligible for inclusion, participants had to self-report current experiences of CBS, have impaired vision (regardless of the cause), be 18 years or older and not report any cognitive impairments or a history of Parkinson’s disease. In addition, participants needed to have completed the baseline questionnaire in the initial study. A formal clinical diagnosis of CBS was not required, and all types of visual hallucinations (simple and complex) were accepted. The unit of participation was the individual, and for the purposes of the present pre–post analysis, all included participants received the educational podcast intervention; no allocation to separate study conditions was undertaken.

### Podcast development and delivery

The podcast was developed by a multidisciplinary team, including researchers (BH, DT and TC), a psychiatrist, a patient with lived experience of CBS, and Wordbird, a UK-based healthcare communications agency. Wordbird recorded the podcasts pro bono, with an experienced narrator ensuring a smooth delivery. Recordings took place at Wordbird’s offices and a hospital office using professional-grade equipment. Editing was handled by Wordbird, while the research team and colleagues at City St George’s, University of London conducted post-production checks to ensure clarity and alignment with study aims.

As part of a larger study of the impact of educational podcasts on behaviour change in people with CBS, two similar versions of the podcast were produced and shared with participants, whereby one differed only in a brief segment at the end. In a previously published study, these two podcasts were compared to determine whether the inclusion of this additional closing statement influenced behavioural responses to CBS hallucinations.^17^ The study found no statistically significant difference between the two podcasts in altering behaviour. In the present study, data from both podcast versions were combined to examine the broader effect of an educational podcast intervention on emotional wellbeing and attitudes toward hallucinations.

**Figure 1.**
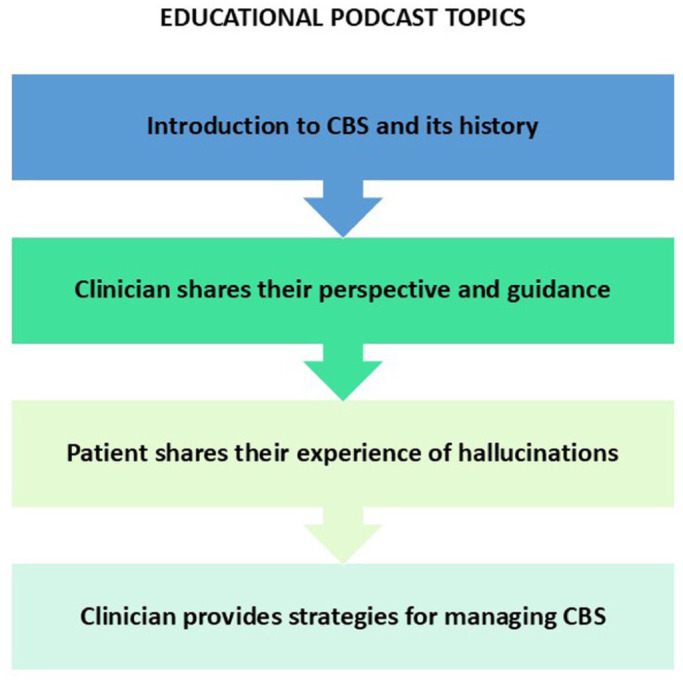
Flowchart illustrating topics covered by podcast in sequential order.

Both versions were ~15 min long and contained identical educational content, covering CBS and its historical background, insights from a psychiatrist, and a patient’s personal experiences. The podcasts also presented identical management strategies, including adjusting lighting, using distraction techniques, and implementing stress management tactics (see Figure 1). However, an additional closing statement encouraging listeners to actively engage with their hallucinations as a potential strategy for mitigating episodes was included in one version. Aside from this final encouragement, both versions were structurally and thematically identical. Given this high degree of similarity, emotional impacts were assessed across all participants as a single cohort for the present report. For simplicity, the podcast will be referred to in the singular throughout this report, as both versions were nearly identical in content and purpose.

**Figure 2.**
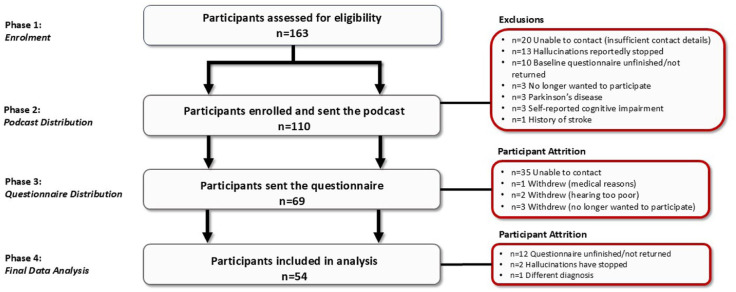
Flow diagram of protocol followed and participant attrition.

The podcast was shared with the participants via online link, CD, USB stick or MP3 player, compatible with audiobook players (depending on preference), by a researcher from City University of London (BH). Participants were asked to listen to the podcast at least once, but were encouraged to listen to it as many times as they wanted. They were given a 3-week window with the podcast at home before being sent the follow-up questionnaire. This window of time with the intervention was deemed sufficient, as >90% of recruited participants reportedly experience CBS hallucinations at a minimum of once a week. See Figure 2 for the flow diagram of the study protocol followed.

### Questionnaires and study outcome measures

The baseline questionnaire administered as part of a broader study on the psychosocial impact of CBS included demographic and clinical information, as well as characteristics of and attitudes towards hallucinations. The participants were presented with the statement ‘*Charles Bonnet Syndrome has the following effect on my life*’ and were asked to rate their experience on a 5-point Likert scale from ‘a very negative affect’ to ‘a very pleasant effect’.^11^ Levels of positive and negative affect (a measure of mood or emotional state) were assessed using PANAS (with scores ranging from 10 to 50, with lower scores representing lower levels of negative affect).^18^ The PANAS has demonstrated good internal consistency (Cronbach’s α > 0.80). These data, reported previously^10^ was used to characterise these participants at baseline (i.e. before the podcast).

The follow-up questionnaire was sent to all participants after having access to the podcast for 3-weeks. As in the baseline questionnaire, participants were asked about the reported impact that CBS has on their life and levels of negative affect were also assessed again using the PANAS. These were the two main outcome measures of this study. While the survey used was part of a broader study and featured additional questions, the focus of this present report is on these specific outcome measures. The full baseline questionnaire and the follow-up questionnaire are available in the Supplemental Material.

### Power calculation

As this is the first study to examine the impact of educational podcasts on emotional wellbeing in individuals with CBS, there were no existing data to estimate an expected effect size. In our previously published study comparing two podcast versions, a pragmatic sample size of 50 participants per arm (100 participants in total) was determined, providing 80% power to detect a standardised mean difference of 0.6 between groups at a two-sided significance level of 0.05.^16,17^ Given the pooling of podcast data in the present analysis, this sample size provides adequate power to detect meaningful changes in the combined cohort.

### Statistical analysis

All data analyses were performed in R version 4.2.2 (http://www.r-project.org/) under R Studio (RStudio, Boston, MA, USA), including use of the ggplot2 package. Baseline measures of negative affect and self-reported impact of hallucinations were established and compared before and after receiving the podcast. Statistical analysis was conducted to compare participants’ responses to the questionnaires. Descriptive statistics were used for clinical and hallucination characteristic data. The non-parametric Wilcox test was used to measure the difference between the non-normally distributed outcome measures: perceived impact of CBS and level of negative affect. Analyses were conducted at the level of the individual participant, with pre- and post-intervention responses paired within participants. The mean negative affect score from normative data collected by Crawford and Henry (2004) from 1003 members of the general public (n466 men, n537 women) with a mean age of 43 years (range 18–91 years) was 14 (SD ± 5.9), and this was used as a normative reference limit.^19^ This approach has been documented in a previous study.^10^ 95% Confidence Intervals (CI) are reported for proportion data. Analyses included all participants who completed both baseline and follow-up questionnaires, with complete data for all outcomes.

## Results

### Demographics and clinical characteristics

Fifty-four people with CBS (74% female; *n* = 43 aged >65 years) were deemed eligible for inclusion in the study, received the podcast and completed the final questionnaire (see Table 1). For details of the study design and participant attrition with associated reasons, see Figure 2. The most common vision impairment in the cohort was AMD (*n* = 30) and 78% had binocular vision loss. See Table 1 for details.

**Table 1.** Demographics and clinical characteristics of population.

Demographic and clinical characteristics	*N*
18–35 years	1
36–50 years	4
51–65 years	6
Over 65 years	43
Female	40
Male	14
Age-related macular degeneration	34
Glaucoma	8
Retinitis Pigmentosa	3
Cataracts	3
Tumour (brain or ocular)	3
Other*	8
Monocular impairment	12
Binocular impairment	42

As some have binocular visual impairment, differing diagnosis in each eye may be recorded.

* ‘Other’ diagnosis include stroke (*n* = 2), Leber’s hereditary optic neuropathy (*n* = 1), diabetic retinopathy (*n* = 1), hydrocephalus (*n* = 1), Pseudo Xanthoma Elasticum (*n* = 1), retinal vein occlusion (*n* = 1), hemianopia (*n* = 1) and unreported (*n* = 2).

### Participant attrition

Participants were recruited between March 2022 and March 2023, with a target sample size of 100. A total of 110 individuals were enrolled. However, approximately half of the participants were lost to follow-up, with the highest attrition (62%) occurring between receiving the educational podcast and completing the questionnaire (see Figure 2). Dropout rates were notably higher among those who provided only an email address and received study materials and the podcast link via email. In contrast, participants who shared a phone number or postal address had lower attrition rates, as they were directly contacted by a member of the research team (BH). The 54 participants included in the final analysis did not differ significantly from the 110 initially enrolled in age, gender, or hallucination frequency and duration.

### Hallucination characteristics

A large proportion of participants (*n* = 22 [41%; 95% CI: 28%–55%]) reported hallucinations to last minutes opposed to seconds or hours, and many experienced symptoms almost every day (*n* = 20 [37%; 95% CI: 24%–51%]). The vast majority of participants (*n* = 43 [80%; 95% CI: 66%–89%] experienced complex hallucinations (people, faces, animals, objects etc.) while only 11 participants (20% [95% CI: 10%–34%] experienced simple hallucinations only (colours, flashing lights).

### Changes in perceived impact of hallucinations

**Figure 3.**
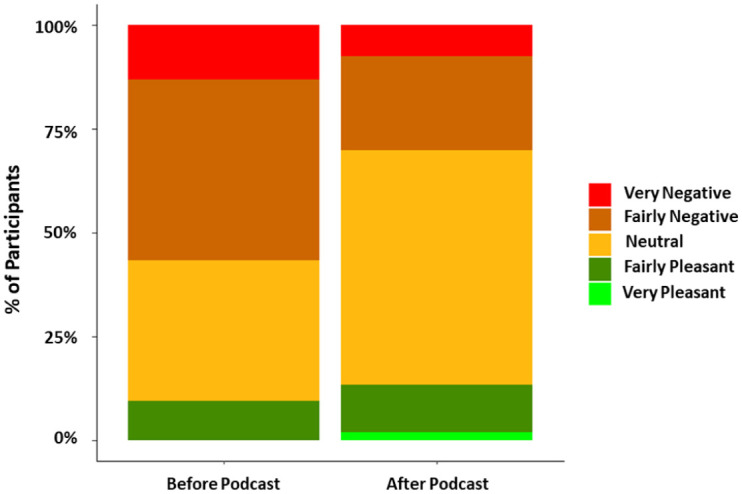
Plot depicting reported impact of CBS on participants’ lives before and after the educational podcast. Prior to the podcast, no one reported the impact of CBS as ‘very pleasant’ on their life. CBS, Charles Bonnet Syndrome.

Before receiving the educational podcast, 30 participants (56% [95% CI: 41%–69%]) classified CBS as having a negative impact on their lives, thus categorising them as having negative-outcome CBS.^11^ After the educational podcast, this number nearly halved to 16 participants (30% [95% CI: 18%–44%]) (see Figure 3). 22 participants (41% [95% CI: 28%–55%]) reported a less negative impact of CBS on their lives compared to before the podcast, while 23 (43% [95% CI: 29%–57%]) reported no change, and eight (15%; [95% CI: 6%–27%]) experienced a worsening impact. The reported impact of CBS was significantly lower after the educational podcast compared to before (*p* < 0.01).

### Changes in levels of negative affect

**Figure 4.**
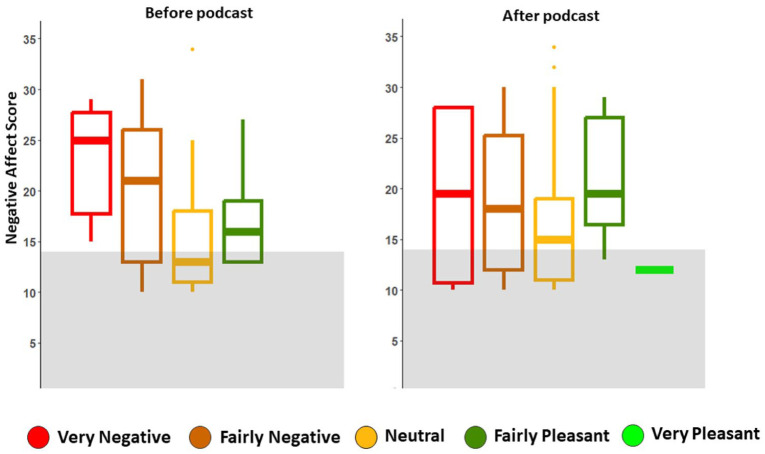
Plots depicting reported levels of negative affect, stratified by reported impact of CBS on participants’ lives, before and after the podcast. The mean negative affect score for *n* = 1003 members of the general public was reported as 14 by Crawford and Henry.^19^ This score is shown by the grey shaded area of the plot. All participants who rated the impact of CBS as ‘very negative’ scored a higher level of negative affect than the mean score of the general public and no one reported the impact of CBS as ‘very pleasant’ on their life prior to the podcast distribution.

Before the educational podcast, the mean negative affect score for participants was 20.37 (SD ± 8.99), where higher scores indicate increased levels of negative affect. This was notably higher than the mean score of the general population, which is 14 (SD ± 5.9).^15^ All participants who rated the impact of CBS as ‘very negative’ exhibited a higher level of negative affect compared to the general population mean prior to receiving the podcast (see Figure 4). After engaging with the educational podcast, participants’ overall negative affect decreased to 18.23 (SD ± 7.87), with a statistically significant improvement in negative affect levels (*p* = 0.02).

## Discussion

This study investigated the impact of an educational podcast on the emotional wellbeing of participants and their reported ratings of CBS impact on their lives. By assessing changes in both emotional state and perceived burden of symptoms, this study aimed to determine whether a podcast could serve as a meaningful tool in improving wellbeing and fostering a more positive outlook for individuals living with CBS. The findings indicated that participants reported statistically significant improvements in emotional wellbeing and a reduction in the perceived negative impact of CBS after listening to the podcast. These findings highlight the potential of educational podcasts as an accessible form of support for people with CBS, offering a means to potentially enhance understanding and emotional resilience. More broadly, this underscores the role of digital health communication in addressing the psychosocial challenges associated with vision impairment.

The significant reduction in negative affect scores and the improved perception of the impact of CBS on participants’ lives indicate that the podcast may have provided valuable emotional support and worked to normalise participants’ experiences. Hearing from experts and individuals with CBS may have validated experiences and reduce feelings of isolation, which aligns with theories of social support as a buffer against stress.^20^ Furthermore, within the framework of Self-Determination Theory (SDT), the engaging and supportive content of the podcast may have enhanced feelings of autonomy, competence, and relatedness, which are crucial for emotional wellbeing.

Prior research has shown that people with CBS report significantly more symptoms of depression and anxiety compared to the general population^21,22^ and a link has been established between poor emotional wellbeing and a perceived more negative impact of CBS.^10^ In addition, vision-related QOL (measured via the National Eye Institute Visual Function Questionnaire 25 [NEI VFQ-25]) has been found to be significantly lower in glaucoma patients with CBS compared to patients without.^23^ The data from this study underscores the importance of recognising the detrimental impact CBS can have on emotional wellbeing. It also highlights the need for interventions such as an educational podcast to address the negative effects associated with this condition.

While most participants reported improvements in the perceived impact of CBS, a small proportion of participants indicated a worsening of symptoms following the podcast. Given the brief, educational nature of the intervention, it is unlikely that the podcast itself triggered these changes. Instead, they may reflect normal fluctuations in CBS symptomology or heightened awareness and attention to visual hallucinations immediately after engaging with the content. These observations highlight the heterogeneous nature of CBS experiences.

Given the positive emotional benefits found following listening to the educational podcast, the digital tool may be valuable in health communication for other eye-related conditions. They can provide accessible, on-demand education and support for visually impaired populations. Yet, to the authors’ knowledge, there is no existing literature on the use of education podcasts in the field of vision research. Limited research beyond vision shows that educational podcasts positively impact learning, self-efficacy, and attitudes across various subjects.^14^ Dual-Process Theory of Persuasion suggests that podcasts engage both systematic processing (factual learning) and heuristic processing (emotional learning), making the information both compelling and memorable.^24^ Using narratives to convey health-related information can assist with helping individuals process new knowledge and overcome resistance to certain health behaviours.^25^ Real-life experiences presented within a story-like context are harder to dismiss^26^ and have been found to evoke stronger emotional responses compared to just presenting facts.^27^

The number of people listening to podcasts is on the rise, with the estimated number of listeners in the United Kingdom rising from 8.99 to 15.61 million from 2017 to 2020.^28^ Healthcare providers could integrate podcasts into routine care. Podcasts could be recommended as supplementary resources to patients, especially for conditions requiring psychological support. Additionally, there is a drive for healthcare providers to make better use of digital technology to improve the quality and efficiency of services.^29^ This is particularly important in the United Kingdom, when the National Health Service (NHS) is over-burdened^30^ and ophthalmology departments are strained.^31^ A recent survey by Ucan Gunduz et al. highlighted that ophthalmologists’ limited awareness of CBS and the lack of routine inquiry about hallucination symptoms in patients with low vision may contribute to substantial underdiagnosis.^32^ This highlights the need for practical educational strategies to improve patient care. Educational podcasts offer a scalable approach to complement clinician counselling, helping normalise symptoms, reduce distress and address gaps in routine clinical practice.

While our findings suggest that an educational podcast may positively influence emotional wellbeing and the perceived impact of CBS, future work should explore how this intervention compares to other educational formats, such as written materials or in-person support. These alternatives may yield similar benefits, though they may be less accessible for individuals with visual impairments. In addition, although our study identified short-term improvements, it remains unclear whether these effects are sustained over time. Longitudinal research is therefore needed to evaluate these changes and to determine whether ongoing or repeated exposure to such content might enhance or maintain its benefits.

To the author’s knowledge, this is the first study to investigate the impact of a podcast on emotional wellbeing in people with CBS. A key strength of this study is that most participants were already engaged with CBS support networks, as they were mostly recruited from charities, suggesting they were more informed than the general CBS population. The fact that the podcast still significantly reduced negative affect highlights its value beyond education by validating experiences and fostering emotional resilience. This suggests an even greater potential impact for those newly diagnosed or less familiar with CBS. Furthermore, changes in vision-related health are unlikely over the short 3-week study period, supporting that observed outcomes reflect the podcast rather than fluctuations in health status. Another of the study’s strengths includes the use of previously validated questionnaires, which have undergone rigorous testing to ensure reliability and validity, enhancing the quality and credibility of this study and facilitating comparability with existing research. Importantly, the involvement of PPI during the study design of the questionnaire and podcast ensured that the outcomes were relevant and meaningful to those directly affected by the research. Lastly, a further strength of this study is that the educational podcast will be made freely available. This ensures wider accessibility, particularly for the charities involved in recruitment, as well as for anyone seeking support or information.

This study also had limitations. For example, due to the study design, causality cannot be inferred as no control group was included, but the observed improvements in negative affect and attitude towards hallucinations suggest that the podcast likely had a positive effect. Another limitation of the study was participant attrition, which was notably higher among those invited via email compared to more direct methods such as phone calls or letters. Emails are more easily overlooked or filtered into spam, potentially reducing engagement. This may have introduced selection bias, as participants who remained could differ from those who dropped out in engagement or responses. Requesting participants to provide a phone number alongside an email address may have helped mitigate attrition by enabling more direct and personalised follow-up. Furthermore, the cohort examined was predominantly female. In a recent systematic literature review of CBS literature in relation to AMD, it was concluded from 18 studies of >4000 participants that female gender was associated with CBS in people with AMD; therefore, it is perhaps unsurprising our AMD-dominant cohort reflected this greater prevalence.^33^ Lastly, the absence of ethnicity data in this study limits the generalisability of the results. To address this limitation, future studies should prioritise working with ethnically diverse populations and collecting ethnicity data.

In conclusion, the educational podcast was associated with improvements in emotional wellbeing, including a reduction in negative affect and the perceived burden of CBS. These findings suggest that educational podcasts may serve as a valuable tool for supporting the emotional health of individuals with CBS. This study contributes to the growing body of evidence supporting innovative, accessible health communication strategies. Future research should focus on evaluating the long-term impact of such interventions and comparing the usefulness of the intervention on different demographics.

## Supplemental Material

sj-pdf-1-oed-10.1177_25158414261446576 – Supplemental material for The impact of an educational podcast on emotional wellbeing and attitudes towards Charles Bonnet syndrome hallucinations Supplemental material, sj-pdf-1-oed-10.1177_25158414261446576 for The impact of an educational podcast on emotional wellbeing and attitudes towards Charles Bonnet syndrome hallucinations by Bethany E. Higgins, Deanna J. Taylor, Sonali Dave, Sarah Sowerby, David P. Crabb and Tamsin Callaghan in Therapeutic Advances in Ophthalmology

## Declarations

**Ethics approval and consent to participate:** This study was performed in line with the principles of the Declaration of Helsinki. Informed written consent was obtained from participants included in the study. For those who were assessed over the telephone, verbal consent was audio-recorded and approved by the Ethics Committee of City St George’s, University of London (05062023/ETH2223-2255).

**Consent for publication:** Not applicable. No identifiable participant data are included in this manuscript.

**Availability of data and materials:** The datasets generated and analysed during the current study are not publicly available due to the risk of participant identification, given the small sample size and the nature of the consent provided. Data are available from the corresponding author* on reasonable request and in accordance with ethical and data protection guidelines.

## References

[1] Teunisse RJ Cruysberg JR Hoefnagels WH , et al. Visual hallucinations in psychologically normal people: Charles Bonnet’s syndrome. Lancet 1996; 347: 794–797. 8622335 10.1016/s0140-6736(96)90869-7

[2] Carpenter K Jolly JK Bridge H. The elephant in the room: understanding the pathogenesis of Charles Bonnet syndrome. Ophthalmic Physiol Opt 2019; 39: 414–421. 31591762 10.1111/opo.12645

[3] Grasso E Bonomo G Certo F , et al. Visual hallucinations in neurosurgery: a systematic review and two case insights into Charles Bonnet Syndrome. Brain and Spine. Epub ahead of print 1 January 2025. DOI: 10.1016/j.bas.2025.104257. PMC12139488 40476156

[4] Gordon KD. Prevalence of visual hallucinations in a national low vision client population. Can J Ophthalmol 2016; 51: 3–6. 26874151 10.1016/j.jcjo.2015.10.006

[5] Christoph SEG Boden KT Siegel R , et al. The prevalence of Charles-Bonnet syndrome in ophthalmic patients: A systematic review and meta-analysis. Brain Res Bull. Epub ahead of print 1 April 2025. DOI: 10.1016/j.brainresbull.2025.111282. 40049460

[6] Taylor DJ Hobby AE Binns AM , et al. How does age-related macular degeneration affect real-world visual ability and quality of life? A systematic review. BMJ Open 2016; 6: e011504. 10.1136/bmjopen-2016-011504 PMC5168634 27913556

[7] Gothwal VK Mandal AK. Quality of life and life satisfaction in young adults with primary congenital glaucoma. Ophthalmol Glaucoma 2021; 4: 312–321. 33002642 10.1016/j.ogla.2020.09.015

[8] Scott IU Schein OD Feuer WJ , et al. Visual hallucinations in patients with retinal disease. Am J Ophthalmol 2001; 131: 590–598. 11336933 10.1016/s0002-9394(01)00833-9

[9] Jones L Ditzel-Finn L Potts J , et al. Exacerbation of visual hallucinations in Charles Bonnet syndrome due to the social implications of COVID-19. BMJ Open Ophthalmol 2021; 6: 670. 10.1136/bmjophth-2020-000670 PMC7880118 33628948

[10] Higgins B Taylor D Crabb D , et al. Emotional well-being in Charles Bonnet syndrome: exploring associations with negative affect, loneliness and quality of life. Ther Adv Ophthalmol . https://journals.sagepub.com/doi/10.1177/25158414241275444 (2024, accessed 24 February 2025). 10.1177/25158414241275444 PMC11440537 39351142

[11] Cox TM ffytche DH. Negative outcome Charles Bonnet Syndrome. Br J Ophthalmol 2014; 98: 1236. 24825847 10.1136/bjophthalmol-2014-304920 PMC4145458

[12] Kressin NR Spiro A Skinner KM. Negative affectivity and health-related quality of life. Med Care 2000; 38: 858–867. 10929997 10.1097/00005650-200008000-00009

[13] Turner-McGrievy G Kalyanaraman S Campbell MK. Delivering health information via podcast or web: media effects on psychosocial and physiological responses. Health Commun 2013; 28: 101–109. 22420785 10.1080/10410236.2011.651709 PMC4860808

[14] McNamara SWT Brian A Bittner M. Content acquisition podcasts and preservice physical educators’ knowledge and self-efficacy toward teaching students with visual impairments. J Teaching Phys Educ 2021; 41: 356–363.

[15] Robins B Delaney T Maher C , et al. Podcasts as a tool for promoting health-related behaviours: a scoping review. Digit Health 10: 20552076241288630. 10.1177/20552076241288630 PMC11472369 39403714

[16] Des Jarlais DC Lyles C Crepaz N. Improving the reporting quality of nonrandomized evaluations of behavioral and public health interventions: the TREND statement. Am J Public Health 2004; 94: 361–366. 14998794 10.2105/ajph.94.3.361 PMC1448256

[17] Higgins BE Taylor DJ Dave S , et al. Podcast-driven insights into Charles Bonnet Syndrome: Impact on self-management strategies and communication. Digit Health. Epub ahead of print 1 November 2025. DOI: 10.1177/20552076251382830. PMC12627372 41268190

[18] Watson D Clark LA Tellegen A. Development and validation of brief measures of positive and negative affect: the PANAS scales. J Pers Soc Psychol 1988; 54: 1063–1070. 3397865 10.1037//0022-3514.54.6.1063

[19] Crawford JR Henry JD. The Positive and Negative Affect Schedule (PANAS): Construct validity, measurement properties and normative data in a large non-clinical sample. Br J Clin Psychol 2004; 43: 245–265. 15333231 10.1348/0144665031752934

[20] Cohen S Wills TA. Stress, social support, and the buffering hypothesis. Psychol Bull 1985; 98: 310–357. 3901065

[21] Jackson ML Bassett K Nirmalan PK. Charles Bonnet hallucinations: natural history and risk factors. Int Congr Ser 2005; 1282: 592–595.

[22] Menon GJ Rahman I Menon SJ , et al. Complex visual hallucinations in the visually impaired: the charles bonnet syndrome. Surv Ophthalmol 2003; 48: 58–72. 12559327 10.1016/s0039-6257(02)00414-9

[23] Randeblad P Singh A Peters D. Charles Bonnet syndrome adversely affects vision-related quality of life in glaucoma patients. Ophthalmol Glaucoma. Epub ahead of print 2023. DOI: 10.1016/j.ogla.2023.07.001. 37429533

[24] Chaiken S. Heuristic versus systematic information processing and the use of source versus message cues in persuasion. J Pers Soc Psychol 1980; 39: 752–766.

[25] Kreuter MW Holmes K Alcaraz K , et al. Comparing narrative and informational videos to increase mammography in low-income African American women. Patient Educ Couns 2010; 81: S6–S14. 10.1016/j.pec.2010.09.008 PMC3146295 21071167

[26] Knowles ES Linn JA. Resistance and persuasion. Resistance and Persuasion 2003; 1–337.

[27] Kearns C Kearns N. The role of comics in public health communication during the COVID-19 pandemic. J Vis Commun Med 2020; 43: 139–149. 32643470 10.1080/17453054.2020.1761248

[28] Podcasts in the United Kingdom (UK) | Statista, https://www.statista.com/study/78479/podcasting-in-the-uk/ (accessed 14 April 2025).

[29] NHS Long Term Plan » The NHS Long Term Plan, https://www.longtermplan.nhs.uk/publication/nhs-long-term-plan/ (accessed 30 January 2023).

[30] Public perceptions of the NHS and social care: performance, policy and expectations – The Health Foundation, https://www.health.org.uk/publications/long-reads/public-perceptions-performance-policy-and-expectations (accessed 10 January 2023).

[31] Hospital Outpatient Activity 2021-22 – NDRS, https://digital.nhs.uk/data-and-information/publications/statistical/hospital-outpatient-activity/2021-22 (accessed 6 January 2023).

[32] Ucan Gunduz G Yalcinbayir O Gelisken O . Awareness of Charles Bonnet syndrome among ophthalmologists: a survey study. Turkey Int Ophthalmol 2025; 45: 207–207. 40418493 10.1007/s10792-025-03575-6

[33] Niazi S Krogh Nielsen M , et al. Prevalence of Charles Bonnet syndrome in patients with age-related macular degeneration: systematic review and meta-analysis. Acta Ophthalmol 2020; 98: 121–131. 31654492 10.1111/aos.14287

